# Human papillomavirus infections among women with cervical lesions and cervical cancer in Yueyang, China: a cross-sectional study of 3674 women from 2019 to 2022

**DOI:** 10.1186/s12985-023-02221-w

**Published:** 2023-11-02

**Authors:** Min Zeng, Xiaoyun Zhang, LiLi He, Xin Liu, Huawen Liu, Rui Deng, Bo Qiu, Fang Liu, Hang Xiao, Quanlv Li, Wen Li, Chongmei Liu, Yangqing Ge

**Affiliations:** 1 Department of Gynecology, Yueyang People’s Hospital, Yueyang Hospital Affiliated to Hunan Normal University, Chongmei Liu. 263, Baling East Road, Yueyang, 414000 Hunan China; 2 Pre-hospital Emergency Center, Yueyang Central Hospital, Yueyang, 414000 Hunan China; 3 Department of Pathology, Miluo People’s Hospital, Yueyang, 414000 Hunan China; 4 Department of Pathology, Pingjiang County Maternal and Child Health Center, Yueyang, 414000 Hunan China; 5 Department of Clinical Laboratory, Yueyang People’s Hospital, Yueyang Hospital Affiliated to Hunan Normal University, Yueyang, 414000 Hunan China; 6 Department of Pathology, Miluo Maternal and Child Health Center, Yueyang, 414000 Hunan China

**Keywords:** Cervical lesions, Cervical cancer, Human papillomavirus, Genotypes

## Abstract

**Purpose:**

To investigate the distribution of the incidence and genotypes of human papillomavirus (HPV) among women with cervical cancer (CC) and precancerous cervical lesions in Yueyang City, China, to develop prevention and control strategies for CC.

**Methods:**

A total of 3674 patients with cervical lesions and cervical cancer who attended 7 hospitals in Yueyang City between September 2019 and September 2022 were included. They included 1910 cervical intraepithelial neoplasia (CIN) I, 718 CIN II, 576 CIN II and 470 CC, respectively. The HPV genotyping of the above patients was detected by Real time-PCR in the laboratory department of each hospital.

**Results:**

The total HPV prevalence was 74.69% (95% CI 73.28–76.09%) in 3674 patients. The incidence of high- and low-risk HPV was 73.46% and 7.21%, respectively. The prevalence of HPV in CIN I, CIN II, CIN III, and invasive CC (ICC) groups was 66.65% (1273/1910, 95% CI 64.53–68.77%), 80.78% (580/718, 95% CI 77.89–83.67%), 83.88% (483/576, 95% CI 80.84–86.87%), and 86.81% (408/470, 95% CI 83.74–89.88%), respectively. The top three HPV subtypes in ICC are HPV16, HPV52, and HPV58. The prevalence of HPV 16 increased with increasing disease severity, with this genotype being present in 12.57%, 20.89%, 36.98%, and 50.85% of CIN I, CIN II, CIN III, and ICC cases, respectively (*p* < 0.001). Single HPV infection was predominant in cervical lesions, with a prevalence of 48.50% (95% CI 46.89–50.12%). The HPV prevalence varied by age, being highest among women with ICC, CIN I, CIN II and CIN III aged ≥ 60 years, 50–59 years, 40–49 years, and 40–49 years, respectively.

**Conclusion:**

The prevalence of HPV in patients with cervical lesions in Yueyang City was very high, with HPV 16, 52, 58, 53, and 51 being the five most common HPV genotypes in patients with cervical lesions.

## Background

Cervical cancer (CC) is the second most prevalent malignancy in women, with 600,000 new cases and over 340,000 related deaths reported worldwide in 2020. Eighty-five percent of CC cases occurred in developing countries [1]. China remains a country with a high incidence and mortality rate of CC, with nearly 110,000 new cases and approximately 60,000 related deaths in 2020 [2].

Human papillomaviruses (HPVs) are the most common sexually transmitted viruses [3] and are divided into two groups, high-risk (HR)-HPV and low-risk (LR)-HPV, HR-HPV including HPV16, 18, 31, 33, 35, 39, 45, 51, 52, 56, 58, 59, 68, 73 and HPV82 [4]. Seventy-five percent of women are infected with HPV in their lifetime; 90% of these infections resolve spontaneously [5]. Persistent HPV infection may significantly increase the risk of the progression of cervical intraepithelial neoplasms (CINs) to CC [6–9].

CIN is a precancerous lesion that is histologically classified as CIN I, CIN II, or CIN III. Persistent HPV infection may cause CIN II and CIN III to progress to CC. Therefore, primary and secondary prevention of CC has become a focus for CC treatment. Currently, three HPV vaccines have been approved worldwide and have achieved great success in developed countries, 9-valent human papillomavirus vaccine is more than 90% effective in preventing precancerous cervical lesions and cervical cancer [10, 11]. However, only a very small number of people have received these vaccines in China [12]. Moreover, the distribution of HPV genotypes in cervical lesions in China differs from that in the rest of the world. Therefore, in this study, we investigated the distribution of the incidence and genotypes of HPV among women with cervical lesions in Yueyang City, China, to develop prevention and control strategies for CC in the region.

China's cervical cancer screening rate is not high, and the screening rate varies greatly among different regions [13]. The total HPV prevalence in the general female population aged 20 years and above is 15.0%, with common types including HPV52, 58, 53, 16, 51, and the prevalence of HPV infection is higher in the central and western regions than in the eastern regions [14]. Yueyang city is located in the center of China, and in our previous study, the total HPV prevalence in the female population of Yueyang city was 20.5%, with the top five HPV types being HPV52, 16, 58, 53, and 81 [15]. In a study as early as 2012, it was found that the prevalence of histologically confirmed CIN1, CIN2, and CIN3 and above lesions in China was 3.1%, 1.3%, and 1.2%, respectively [16]. In a MATE analysis in 2018, it was concluded that the prevalence of high-risk HPV infection in the population of women with cytologically normal cervical uterine cervix, LSIL, HSIL, and invasive carcinoma in China was 15.6%, 69.8%, 86.0% and 88.7% respectively [17]. There is no relevant study in Yueyang City that can illustrate the relationship between HPV infection and different levels of cervical lesions in the female population.

## Materials and methods

### Participants and methods

#### Study participants

**Fig. 1:**
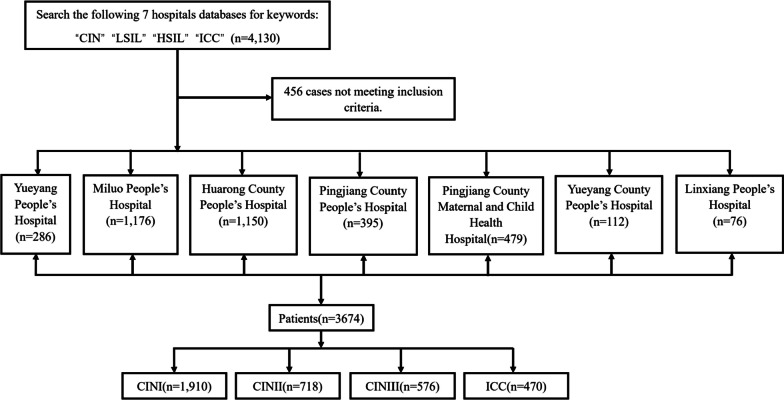
Screening flowchart. CIN, Cervical Intraepithelial Neoplasia; LSIL, Low-grade Squamous Intraepithelial Neoplasia; HSIL, High-grade Squamous Intraepithelial Neoplasia; ICC, Invasive Cervical Cancer

The clinical data of 3674 patients with cervical lesions admitted to 7 hospitals in Yueyang City from September 2019 to September 2022 were selected for a cross-sectional study (Yueyang People’s Hospital, Huarong County People’s Hospital, Pingjiang County People’s Hospital, Miluo City People’s Hospital, Pingjiang County Maternal and Child Health Center, Yueyang County People’s Hospital, Linxiang City People’s Hospital) (Fig. 1). Liquid-based cytology and HPV-DNA testing were used for CC screening. If abnormalities were detected, colposcopic biopsy was performed to diagnose cervical lesions as CIN I, CIN II, CIN III, or invasive CC (ICC). The inclusion criteria were as follows: patients with a confirmed diagnosis of cervical lesions by cervical biopsy; Patients with cervical lesions treated for the first time; patients who underwent liquid-based cytology and HPV-DNA testing; patients with no sexual activity or vaginal medication in the past 48 h; and patients without a history of pelvic radiotherapy. Patients who were pregnant, breastfeeding, had inflammation of the reproductive tract, history of cervical treatment, history of cervical vaccination, or concomitant immunodeficiency were excluded.

### Histological examination and grouping

A colposcopic biopsy of suspicious cervical lesions was performed by gynecologists on an outpatient basis. Histopathologic diagnosis was made by two or more pathologists. Pathological classification was performed in accordance with the 2014 World Health Organization classification criteria [18]: precancerous cervical lesions were classified as low-grade squamous intraepithelial lesions, high-grade squamous intraepithelial lesions, and CC. Since some patients were enrolled before the pathology departments of the 7 hospitals adopted the SIL diagnostic system, the CIN diagnostic system was still used for data processing in this study.

### Methods

#### Specimen collection

The gynecologist rotates a sampling brush five times at the ectocervix to collect a sample of exfoliated cervical cells for HPV DNA testing. All participants provided written informed consent prior to the health screening. The study was approved by the Institutional Review Board of Yueyang Second People’s Hospital (currently renamed Yueyang People’s Hospital) (number: 2019003).

#### HPV-DNA testing

The HPV genotyping test kits applied by Yueyang People’s Hospital, Miluo People’s Hospital, and Yueyang County People’s Hospital were provided by 21 HPV GenoArray Diagnostic Kit (HBGA-21PKG) (Hybribio, China, Guangdong), the HPV test kits applied by Pingjiang People’s Hospital, Pingjiang Maternal and Child Health Hospital, Hualong People’s Hospital were provided by 23 HPV Genotyping Panel kit from (CFDA: 20193401918) (Yanengbio, China, Shenzhen), and the HPV test kits applied by Linxiang People’s Hospital were provided by 26 HPV Genotyping Panel kit from (CFDA: 20173404697) (TELLGEN Life Science, China, Shanghai). The sensitivity and specificity of these kits were higher than 95% compared to FDA approved kit.

Cervical smear samples were stored in appropriate sample transport media. DNA extraction, amplification, and hybridization were performed according to the instructions of each HPV genotyping kit manufacturer. The HPV DNA microarrays in the kits contained 21 specific probes to identify 15 high-risk genotypes (16, 18, 31, 33, 35, 39, 45, 51, 52, 53, 56, 58, 59, 66, and 68) and 6 low-risk genotypes (6, 11, 42, 43, 44, and 81).The PCR amplification step was performed on a T100 Thermal Cycler (Bio-Rad, USA), followed by HPV DNA hybridization reactions on an automated analyzer. Final results were obtained by colorimetric changes on the microarray. Human papillomavirus negative and positive controls provided with the kit were used simultaneously for de-testing in each assay.

#### Colposcopy and pathological diagnosis

Colposcopy was performed in patients with positive HR-HPV or positive cytology. Multisite biopsies were performed at the site of suspected lesions and cervical tissues were classified according to the 2014 World Health Organization classification criteria by two experienced pathologists from each hospital. Based on this criteria, the included cases were reclassified as CINI, CINII, CINIII and CC.

## Statistics

In this study, the prevalence of HPV in cervical lesions was used as the main research indicator, which is known to be 83.83% based on the results of similar published literature [19]. Using the following formula, α = 0.05 (two-sided test) was selected, the permissible error δ = ± 1.3%, *Z*1 − α/2 = 1.96, *p* = 83.83%, *n* = (*Z*1 − α/2δ)^2^*p*(1 − *p*), 3082 cases need to be investigated. Considering the loss follow-up rate of 10–20%, a total of 3674 people were included in the study, which can ensure the accuracy and scientific validity of the results.

SPSS Statistics 23.0 software was used for data analysis. Categorical variables were expressed as frequencies (proportions). Categorical variables were compared using Chi-square or Fisher’s exact probability tests. Pairwise comparison between groups was performed using the Bonferroni method, adjusting for the level of *p* values. *p* values < 0.05 (two-sided) were considered statistically significant.

## Results

### HPV distribution characteristics in patients with cervical lesions

Of the 3674 patients with cervical lesions, 2744 were positive for HPV, giving a prevalence of 74.69% (95% CI 73.28–76.09%). The prevalence of HPV in CIN I, CIN II, CIN III, and ICC groups were 66.65% (1273/1910, 95% CI 64.53–68.77%), 80.78% (580/718, 95% CI 77.89–83.67%), 83.88% (483/576, 95% CI 80.84–86.87%), and 86.81% (408/470, 95% CI 83.74–89.88%), respectively. The total HPV prevalence in patients with precancerous cervical lesions was 72.91% (2336/3204, 95% CI 71.37–74.45%), which was lower than the total HPV prevalence in patients with CC (408/470, 86.81%, 95% CI 83.74–89.88%) (*p* < 0.001). The HPV prevalence was higher in patients with CIN II and III than in those with CIN I (*p* < 0.001). Furthermore, the prevalence increases with increasing disease severity.

The prevalence of HR-HPV and LR-HPV was 73.46% (2699/3674, 95% CI 72.03–74.89%) and 7.21% (265/3674, 95% CI 6.38–8.05%), respectively. The five most common HR-HPV genotypes were HPV 16 (22.92%, 95% CI 21.56–24.28%), 52 (20.14%, 95% CI 18.84–21.44%), 58 (14.59%, 95% CI 13.45–15.73%), 53 (6.78%, 95% CI 5.96–7.59%), and 51 (6.42%, 95% CI 5.63–7.22%), and the most common LR-HPV genotype was HPV 81 (3.13%), followed by HPV 42 (1.47%) and 43 (1.33%). The prevalence of HR-HPV was 65.08% (1243/1910, 95% CI 62.94–67.22%), 79.81% (573/718, 95% CI 76.86–82.75%), 83.51% (481/576, 95% CI 80.47–86.55%), and 85.53% (402/470, 95% CI 82.34–88.72%) in CIN I, CIN II, CIN III, and ICC groups, respectively, with high-risk HPV infection predominating in precancerous cervical lesions and CC.

**Fig. 2:**
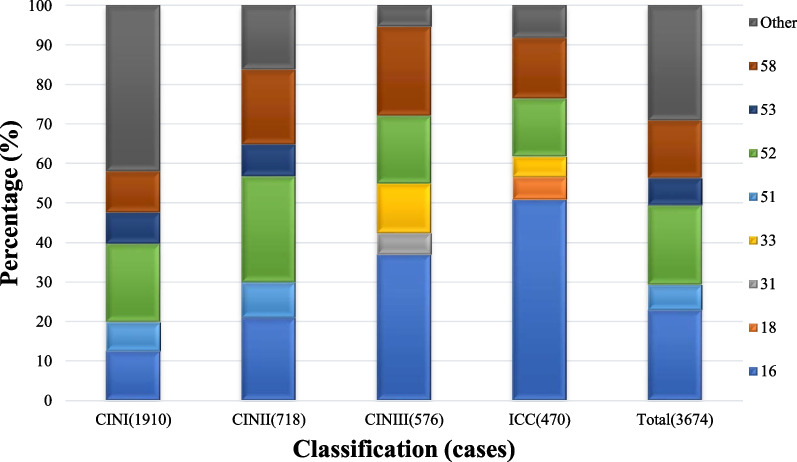
Distribution of HPV infection in different levels of cervical lesions among women in Yueyang. (HPV) 16, 18, 31, 33, 51, 52, 53, 58, Other (%)

HPV 16 (22.92%), 52 (20.14%), 58 (14.59%), 53 (6.78%), and 51 (6.42%) were the five most common HPV genotypes among patients with cervical lesions. The top five genotypes for each cervical lesion grade were HPV 52, 16, 58, 53, and 31 for CIN I; HPV 52, 16, 58, 51, and 53 for CIN II; HPV 16, 58, 52, 33, and 31 for CIN III; and 16, 58, 52, 18, and 33 for ICC. The prevalence of HPV16 was significantly higher in ICC than in CIN I, CIN II and CIN III (*p* < 0.001). The prevalence of in CIN I, CIN II, CIN III, and ICC groups was 12.57%, 20.89%, 36.98%, and 50.85%, respectively. The prevalence of HPV 18 was low in the CIN group but was the fourth highest in the ICC group. The detailed results are shown in Table 1 and Fig. 2.

**Table 1:** The prevalence and genotype distribution of HPV in cervical lesions

HPV	CINI (n = 1910)	CINII (n = 718)	CINIII (n = 576)	ICC (n = 470)	Total (n = 3674)
HPV (–)	637 (33.35)	138 (19.22)	93 (16.15)	62 (13.19)	930 (25.31)
Any HPV	1273 (66.65)	580 (80.78)	483 (83.88)	408 (86.81)	2744 (74.69)
High-risk HPV	1243 (65.08)	573 (79.81)	481 (83.51)	402 (85.53)	2699 (73.46)
16	240 (12.57)	150 (20.89)	213 (36.98)	239 (50.85)	842 (22.92)
52	379 (19.84)	192 (26.74)	99 (17.19)	69 (14.68)	740 (20.14)
58	198 (10.37)	136 (18.94)	130 (22.57)	72 (15.32)	536 (14.59)
53	151 (7.91)	58 (8.08)	20 (3.47)	20 (4.26)	249 (6.78)
51	139 (7.28)	65 (9.05)	18 (3.13)	14 (2.98)	236 (6.42)
68	94 (4.92)	38 (5.29)	15 (2.60)	9 (1.91)	156 (4.25)
33	76 (3.98)	45 (6.27)	72 (12.50)	24 (5.11)	217 (5.91)
39	100 (5.24)	38 (5.29)	16 (2.78)	19 (4.04)	173 (4.71)
56	79 (4.14)	26 (3.62)	18 (3.13)	14 (2.98)	138 (3.76)
18	108 (5.65)	41 (5.71)	17 (2.95)	27 (5.74)	194 (5.28)
66	61 (3.19)	17 (2.37)	4 (0.69)	4 (0.85)	86 (2.34)
59	63 (3.30)	18 (2.51)	3 (0.52)	11 (2.34)	95 (2.59)
31	49 (2.57)	34 (4.74)	31 (5.38)	18 (3.83)	133 (3.62)
35	24 (1.26)	14 (1.95)	6 (1.04)	7 (1.49)	51 (1.39)
45	29 (1.52)	6 (0.84)	8 (1.39)	3 (0.64)	46 (1.25)
Low-risk HPV	151 (7.91)	47 (6.55)	35 (6.08)	32 (6.81)	265 (7.21)
81	64 (3.35)	19 (2.65)	16 (2.78)	15 (3.19)	115 (3.13)
42	32 (1.68)	10 (1.39)	6 (1.04)	6 (1.28)	54 (1.47)
43	27 (1.41)	8 (1.11)	8 (1.39)	6 (1.28)	49 (1.33)
6	19 (0.99)	13 (1.81)	3 (0.52)	5 (1.06)	39 (1.06)
44	6 (0.31)	1 (0.14)	4 (0.69)	1 (0.21)	12 (0.33)
11	15 (0.79)	2 (0.28)	1 (0.17)	2 (0.43)	20 (0.54)

The prevalence of HR-HPV in ICC is significantly different from that of in precancerous cervical lesions (CIN I, χ^2^ = 73.92, *P* < 0.001; CIN II, χ^2^ = 6.33, *P* = 0.012); except CINIII (CIN III, χ^2^ = 0.81, *P* = 0.369)

The prevalence of HPV16 in ICC is significantly higher than that of in precancerous cervical lesions,* P* < 0.001 (CIN I, χ^2^ = 343.92; CIN II, χ^2^ = 115.78; CIN III, χ^2^ = 20.30)

The prevalence of HPV type 16 in ICC is significantly higher than that of other high-risk HPV types, *P* < 0.001 (HPV52, χ^2^ = 139.56; HPV58, χ^2^ = 134.01; HPV18, χ^2^ = 235.65; HPV33, χ^2^ = 244.04)

The prevalence of HPV52 was significantly higher than that of HPV16 in patients with CINI and CINII, *P* < 0.01 (CIN I, χ^2^ = 37.25, *P* < 0.001; CIN II, χ^2^ = 6.77,* P* = 0.009)

### Single and multiple HPV infections in different types of cervical lesions

Cervical lesions were dominated by a single HPV infection, with a prevalence of 48.50% (1782/3674, 95% CI 46.89–50.12%), followed by double and multiple HPV infections with a prevalence of 17.45% (641/3674, 95% CI 16.22–18.67%) and 8.74% (321/3674, 95% CI 7.82–9.65%), respectively. The most multiple infections were caused by nine genotypes. The CIN I, CIN II, CIN III and ICC groups were most commonly characterized by single infections, corresponding to single HPV prevalence rates of 42.20% (806/1910, 95% CI 39.98–44.42%), 49.16% (353/718, 95% CI 45.50–52.83%), 57.29% (330/576, 95% CI 53.24–61.34%) and 62.34% (293/470, 95% CI 57.94–66.74%), respectively. The prevalence of double HPV was 16.60% (317/1910, 95% CI 14.93–18.27%), 20.47% (147/718, 95% CI 17.52–23.43%), 17.88% (103/576, 95% CI 14.74–21.02%), and 15.74% (74/470, 95% CI 12.44–19.05%) in CIN I, CIN II, CIN III, and ICC groups, respectively, and the prevalence of triple and above HPV was 7.85% (150/1910), 11.14% (80/718), 8.68% (50/576), and 8.72% (41/470), respectively (Table 2).

**Table 2:** The prevalence of single and multiple HPV infections in cervical lesions

HPV infection	CINI n (%)	CINII n (%)	CINIII n (%)	ICC n (%)	Total n (%)
Any type	1273 (66.65)	580 (80.78)	483 (83.88)	408 (86.81)	2744 (74.69)
1 HPV subtype	806 (42.20)	353 (49.16)	330 (57.29)	293 (62.34)	1782 (48.50)
2 HPV subtype	317 (16.60)	147 (20.47)	103 (17.88)	74 (15.74)	641 (17.45)
3 HPV subtype	96 (5.03)	54 (7.52)	32 (5.56)	27 (5.74)	209 (5.69)
4 HPV subtype	40 (2.09)	16 (2.23)	14 (2.43)	9 (1.91)	79 (2.15)
5 HPV subtype	14 (0.73)	4 (0.56)	4 (0.69)	3 (0.64)	25 (0.68)
6 HPV subtype	0 (0.00)	4 (0.56)	0 (0.00)	1 (0.21)	5 (0.14)
7 HPV subtype	0 (0.00)	0 (0.00)	0 (0.00)	0 (0.00)	0 (0.00)
8 HPV subtype	0 (0.00)	1 (0.14)	0 (0.00)	1 (0.21)	2 (0.05)
9 HPV subtype	0 (0.00)	1 (0.14)	0 (0.00)	0 (0.00)	1 (0.03)

The prevalence of one HPV subtype in ICC is significantly different from that of in precancerous cervical lesions (CIN I, χ^2^ = 61.57, *P* < 0.001; CIN II, χ^2^ = 19.88, *P* < 0.001); except CINIII (CIN III, χ^2^ = 2.74, *P* = 0.098)

The prevalence of monotypic HPV infection was significantly higher than that of polytypic HPV infection in each group of patients with cervical lesions, *P* < 0.001 (CIN I, χ2 = 135.40; CIN II, χ^2^ = 45.92; CINIII, χ^2^ = 111.69; ICC, χ^2^ = 137.21)

### HPV infection and genotype distribution in patients of different ages

**Fig. 3:**
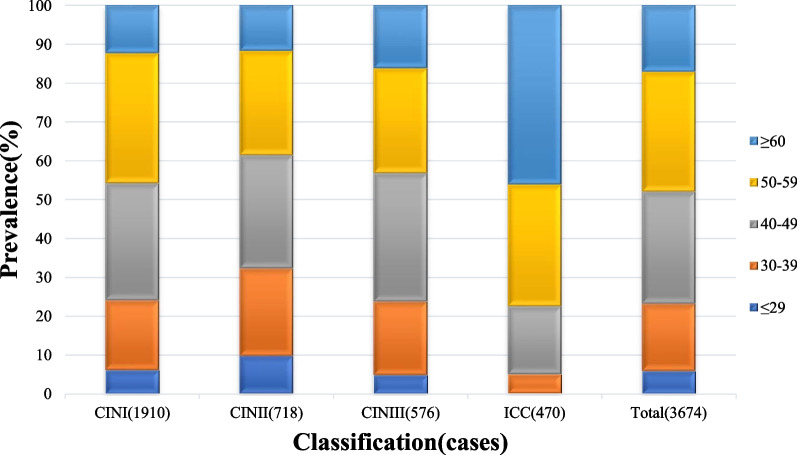
Age stratified distribution of patients with CIN and ICC in Yueyang. ICC, CINIII, CINII, CINI, Total (%)

**Fig. 4:**
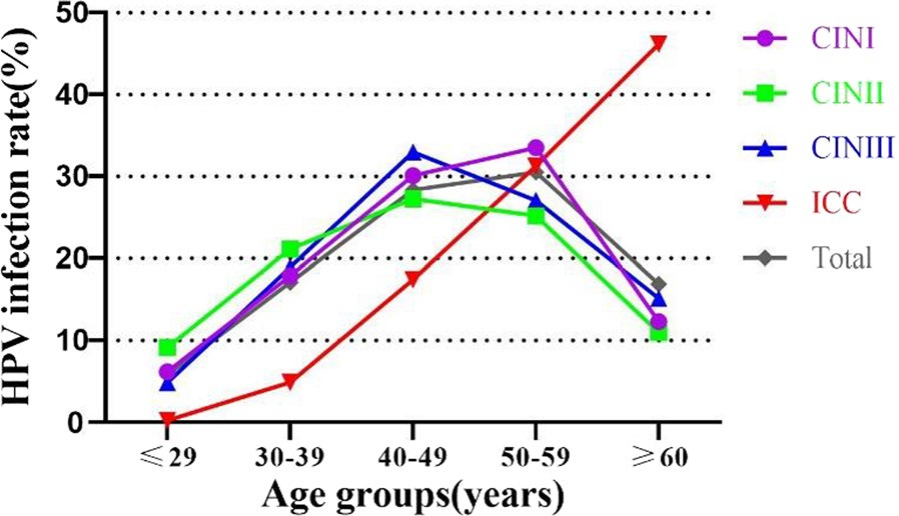
Prevalence of HPV infection in patients with cervical lesions of different age strata in Yueyang

All patients were subdivided into five age groups: ≤ 29 years, 30–39 years, 40–49 years, 50–59 years, and ≥ 60 years. The age-stratified distribution of patients with CIN and ICC in Yueyang is shown in Table 3 and Fig. 3. The prevalence of HPV among the CIN I patients was the highest in the 50–59 years age group (33.51%, 640/1910, 95% CI 31.39–35.63%), which was higher HPV prevalence than the same age group of patients in CIN II, CIN III, and ICC groups. Among patients with CIN II and CIN III, the highest HPV prevalence was found in the age group of 40–49 years (29.11% [209/718, 95% CI 25.78–32.44%]and 32.99% [190/576, 95% CI 29.14–36.84%], respectively), followed by the age group of 50–59 years. In the ICC group, the prevalence gradually increased with age, reaching 46.17% (217/470, 95% CI 41.65–50.69%) in the ≥ 60 years age group (*p* < 0.001) (Table 3, Fig. 4).

**Table 3:** Age distribution of patients with cervical lesions

Age (years)	CINI n (%)	CINII n (%)	CINIII n (%)	ICC n (%)	Total n (%)
≤ 29	118 (6.18)	70 (9.75)	28 (4.86)	1 (0.21)	217 (5.91)
30–39	342 (17.91)	162 (22.56)	109 (18.92)	23 (4.89)	636 (17.31)
40–49	575 (30.10)	209 (29.11)	190 (32.99)	82 (17.45)	1056 (28.74)
50–59	640 (33.51)	193 (26.88)	156 (27.08)	147 (31.28)	1136 (30.92)
≥ 60	235 (12.30)	84 (11.70)	93 (16.15)	217 (46.17)	629 (17.12)
Total	1910 (100)	718 (100)	576 (100)	470 (100)	3674 (100)

In the ICC group, the prevalence of HPV was significantly higher in the ≥ 60 years group than that of in other age groups, *P* < 0.001 (≤ 29 years group, χ^2^ = 278.64; 30–39 years group, χ^2^ = 210.58; 40–49 years group, χ^2^ = 89.39; 50–59 years group, χ^2^ = 21.97)

In the CINI group, the prevalence of HPV was significantly higher in the 50–59 years group than that of in other age groups (≤ 29 years group, χ^2^ = 448.47, *P* < 0.001; 30–39 years group, χ^2^ = 121.72, *P* < 0.001; 40–49 years group, χ^2^ = 5.10,* P* = 0.024; ≥ 60 years group, χ^2^ = 243.15, *P* < 0.001)

In the CINII group, the prevalence of HPV was significantly higher in the 40–49 years group than that of in other age groups (≤ 29 years group, χ^2^ = 85.95, *P* < 0.001; 30–39 years group, χ^2^ = 8.03, *P* = 0.005; ≥ 60 years group, χ^2^ = 67.00, *P* < 0.001); except 50–59 years group (50–59 years group, χ^2^ = 0.88,* P* = 0.347)

In the CINIII group, the prevalence of HPV was significantly higher in the 40–49 years group than that of in other age groups (≤ 29 years group, χ^2^ = 148.48, *P* < 0.001; 30–39 years group, χ^2^ = 29.64, *P* < 0.001; 50–59 years group, χ^2^ = 4.78,* P* = 0.029; ≥ 60 years group, χ^2^ = 44.08, *P* < 0.001)

The five most common genotypes of HPV infection among patients with cervical lesions in different age groups are detailed in Table 4. Among patients with CINI, the five most common genotypes of HPV infection in the age group of 50–59 years were HPV 52 (5.81%), 16 (3.93%), 58 (3.66%), 53 (3.04%) and 51 (2.62%). Among CIN II patients, the most common genotypes of HPV infection in the age group 40–49 years were HPV 52 (7.94%), 16 (5.99%), 58 (4.04%), 18 and 51 (1.95%) and 31 (1.39%). Among patients with CIN III, the five most common genotypes of HPV infection in the age group 40–49 years were HPV 16 (11.81%), 58 (7.12%), 52 (4.86%), 33 (3.82%) and 31 (1.56%). Among the ICC patients, the five most common genotypes of HPV infection in the ≥ 60 years age group were HPV 16 (22.13%), 58 (9.36%), 52 (8.51%), 53 (2.98%), and 51 (2.77%).

**Table 4:** The 5 most common HPV genotypes in different age groups

Age groups (years)	CINI		CINII		CINIII		ICC	
	HPV +	N (%)	HPV +	N (%)	HPV +	N (%)	HPV +	N (%)
≤ 29	52	23 (1.20)	16	21 (2.92)	16	16 (2.78)	16	1 (0.21)
	16	22 (1.15)	52	18 (2.51)	33	5 (0.87)		
	58	11 (0.58)	58	11 (1.53)	52	4 (0.69)		
	51	10 (0.52)	51	7 (0.97)	31	3 (0.52)		
	18,39	9 (0.47)	33	8 (1.11)	58	2 (0.35)		
30–39	52	71 (3.72)	52	44 (6.13)	16	41 (7.12)	16	9 (1.91)
	16	47 (2.46)	16	40 (5.57)	58	18 (3.13)	33,58	5 (1.06)
	58	31 (1.62)	58	37 (5.15)	52	14 (2.43)		
	18	30 (1.57)	39	13 (1.81)	33	13 (2.26)		
	53	27 (1.41)	51,53	11 (1.53)	18,39,51,53	4 (0.69)		
40–49	52	125 (6.54)	52	57 (7.94)	16	68 (11.81)	16	43 (9.15)
	16	71 (3.72)	16	43 (5.99)	58	41 (7.12)	52	17 (3.62)
	58	50 (2.62)	58	29 (4.04)	52	28 (4.86)	58	8 (1.70)
	51	39 (2.04)	18,51	14 (1.95)	33	22 (3.82)	31	6 (1.28)
	68	30 (1.57)	31	10 (1.39)	31	9 (1.56)	18	5 (1.06)
50–59	52	111 (5.81)	52	46 (6.41)	16	56 (9.72)	16	82 (17.45)
	16	75 (3.93)	16	34(4.74)	58	36 (6.25)	58	15 (3.19)
	58	70 (3.66)	58	43 (5.99)	52	29 (5.03)	18	11 (2.34)
	53	58 (3.04)	53	20 (2.79)	33	16 (2.78)	52	10 (2.13)
	51	50 (2.62)	51	21 (2.92)	81	10 (1.74)	33	7 (1.49)
≥ 60	52	49 (2.57)	52	27 (3.76)	58	33 (5.73)	16	104 (22.13)
	58	36 (1.88)	58	16 (2.23)	16	32 (5.56)	58	44 (9.36)
	53	33 (1.73)	53	15 (2.09)	52	24 (4.17)	52	40 (8.51)
	16	25 (1.31)	16,51	12 (1.67)	33	16 (2.78)	53	14 (2.98)
	51	24 (1.26)	33	10 (1.39)	31	12 (2.08)	51	13 (2.77)

In the CIN I group, HPV 52 was the most common genotype across all age groups, followed by HPV 16, which ranked fourth except in the ≥ 60 years age group. In the CIN II group, HPV 52 and HPV 16 were the two most common genotypes in patients under the age of 60 years, and in the CIN III group, HPV 16 was the most common genotype in nearly all age groups, ranking second only in the ≥ 60 years age group. In the ICC group, HPV 16 was the most common genotype across all age groups.

## Discussion

In this study, we analyzed the distribution of the incidence and genotypes of HPV among women with cervical lesions in Yueyang City, China, with an aim of developing prevention and control strategies for CC in the region. We found that the incidence of HPV infection among women with cervical lesions in Yueyang City was very high, and HPV 16, 52, 58, 53, and 51 were the five most common HPV genotypes among these patients.

The most common HPV genotypes in Asia are HPV 16, HPV 52, HPV 58, HPV 18, and HPV 56 [20]. In China, there are differences in HPV infection types among different cities, with the most common genotypes being 52, 58, 16, and 51 in Zhejiang; 16, 52, 58, 53, and 31 in Xinjiang; and 16, 58, 52, 51, and 54 in Shanghai [12, 21, 22]. In the present study, we investigated the prevalence of HPV in 3674 patients with cervical lesions in Yueyang City, and the most common HPV genotypes were HPV 16, 52, 58, 53, and 51.

Persistent HPV infection leads to the development and progression of cervical lesions and CC. In the present study, the incidence of HPV infection in patients with CIN I, CIN II, CIN III, and ICC was 66.65%, 80.78%, 83.88%, and 86.81%, respectively. Li et al*.* [23] reported that the prevalence of HPV infection in CIN I, CIN II+, and CC samples was 59.6%, 84.8%, and 89.9%, respectively. In another study, Lei et al*.* [24] investigated 1664 female patients and found that HPV positivity in these patients increased directly with the severity of cervical lesions (72.4% for CIN I, 81.4% for CIN II, 88.1% for CIN III, and 90.4% for ICC), There are many similar studies [25, 26]. The consistency of these data suggests that HPV positivity increases with the severity of cervical lesions. The overall incidence of HPV in patients with ICC in this study was 86.81%, which is consistent with the global incidence of HPV in CC patients (86–94%) [27].

In the present study, HPV 52, 16, and 58 were found to be the major contributors to CIN I and II, and HPV 16, 58, and 52 were the major contributors to CIN III and ICC, Similar to Shanxi Province [25], Distinguish from Jiangsu and Sichuan [19, 28]. On the whole, these findings are similar to those of previous domestic and international studies. HPV 16 was the predominant subtype, with an incidence of 12.57%, 20.89%, 36.98%, and 50.85% in the CIN I, CIN II, CIN III, and ICC groups, respectively. The incidence increased with the severity of cervical lesions, with HPV 16 being predominant.

HPV 52 is the most prevalent genotype [21, 29–31] in many regions of China and is a major contributor to CC. However, among the 3674 patients in our study, HPV 52 was most common in low-grade squamous intraepithelial lesions of the cervix, specifically ranking first in prevalence in the CIN I and CIN II groups (incidence of 19.84% and 26.74%, respectively) and ranking third in prevalence in the CIN III and ICC group (incidence of 17.19% and 14.68%, respectively). In contrast, HPV 58 ranked third in the CIN I and CIN II groups (incidence of 10.37% and 18.94%, respectively) and second only to HPV 16 in the CIN III and ICC groups (incidence of 22.57% and 15.32%, respectively). These findings are consistent with those of a study of HPV genotype distribution among 7747 women in South Sichuan [32] and 40,311 women in southwest China [33]. Therefore, our findings suggest that HPV 58 is the most oncogenic subtype other than HPV 16 in the Yueyang region. However, among the three vaccines, only the 9-valent vaccine covers HPV 58 and few women received the 9-valent vaccine, emphasizing the importance of vaccinating women in the Yueyang region with the 9-valent vaccine.

HPV 18 has been shown to be the second most common genotype worldwide [34, 35]; however, it had a lower incidence in this study (5.65%, 5.71%, and 2.95% in the CIN I, CIN II, and CIN III groups, respectively; all ranking above fifth, and 5.74% in ICC patients, ranking fourth). Previous studies have suggested that the incidence of HPV 18 infection may be associated with higher rates of adenocarcinoma [36]; however, very few cases in the present study were classified as cervical adenocarcinoma, and further validation of the results in a large-sample study is still required.

Age is one of the most important factors influencing HPV infection, and in the present study, HPV infection in high-grade squamous intraepithelial lesions (CIN II and III) was noted in patients aged 40–49 years. This finding was similar to that in studies from Taizhou and Jiangsu provinces [19, 37]. The age group with the highest prevalence of HPV in the ICC group was the ≥ 60 years group (incidence: 46.17%); this incidence was higher than that in the 50–59 years age group in a study from Taizhou [19], higher than that in the 40–44 years age group in a study from Shanghai and Zhejiang [38], and higher than that in the 41–50 years age group in a study from Jiangsu [37]. This may be related to the increasing attention to CC prevention and treatment in Yueyang; however, the small sample size in the present study cannot be neglected. HPV 16 was the most common genotype across all age groups in the CIN III and ICC groups, except for that in the ≥ 60 years age group in the CIN III group (ranking the second highest in this study). However, in the CIN I and II groups, the most common genotype was HPV 52 across almost all age groups. This result again suggests that the incidence of HPV 16 increases with the increasing severity of cervical lesions.

In conclusion, HPV subtypes are distributed differently in different regions, and the data from the present study supports the use of preventive HPV vaccines in Yueyang. In addition, this study provides important basic data for the prevention and surveillance of HPV infection in the region, which is important for clinical prevention and treatment of CC, prediction of lesion progression, and assessment of outcomes.

## Data Availability

All data generated or analyzed during this study are included in this published article.
